# Effect of *Berberis vulgaris* L. root extract on ifosfamide-induced in vivo toxicity and in vitro cytotoxicity

**DOI:** 10.1038/s41598-020-80579-5

**Published:** 2021-01-18

**Authors:** Shazia Ilyas, Raheela Tabasum, Ali Iftikhar, Mamoona Nazir, Amina Hussain, Aroosha Hussain, Muhammad Sajjad Ali, Farooq Saleem, Uzma Saleem, Matheus Froeyen, Iskandar Abdullah, Muhammad Usman Mirza, Sarfraz Ahmad

**Affiliations:** 1 Department of Biochemistry, Institute of Molecular Biology and Biotechnology (IMBB), University of Lahore, Lahore, 54000 Pakistan; 2 Faculty of Pharmacy, The University of Lahore, Lahore, 54000 Pakistan; 3 Institute of Biochemistry and Biotechnology, University of the Punjab, Lahore, 54000 Pakistan; 4 Riphah Institute of Pharmaceutical Sciences, Riphah International University, Lahore, Pakistan; 5 Department of Pharmaceutical and Pharmacological Sciences, Rega Institute for Medical Research, Medicinal Chemistry, University of Leuven, 3000 Leuven, Belgium; 6 Drug Design and Development Research Group, Department of Chemistry, Faculty of Science, Universiti Malaya, 50603 Kuala Lumpur, Malaysia

**Keywords:** Biochemistry, Cancer

## Abstract

Ifosfamide is a widely used chemotherapeutic agent having broad-spectrum efficacy against several tumors. However, nephro, hepato, neuro cardio, and hematological toxicities associated with ifosfamide render its use limited. These side effects could range from organ failure to life-threatening situations. The present study aimed to evaluate the attenuating efficiency of *Berberis vulgaris* root extract (*Bv*RE), a potent nephroprotective, hepatoprotective, and lipid-lowering agent, against ifosfamide-induced toxicities. The study design comprised eight groups of Swiss albino rats to assess different dose regimes of *Bv*RE and ifosfamide. Biochemical analysis of serum (serum albumin, blood urea nitrogen, creatinine, alanine transaminase, aspartate transaminase, alkaline phosphatase, lactate dehydrogenase, total cholesterol, and triglycerides) along with complete blood count was performed. Kidney, liver, brain, and heart tissue homogenates were used to find malondialdehyde, catalase, and glutathione S-transferase levels in addition to the acetylcholinesterase of brain tissue. The results were further validated with the help of the histopathology of the selected organs. HeLa cells were used to assess the effect of *Bv*RE on ifosfamide cytotoxicity in MTT assay. The results revealed that pre- and post-treatment regimens of *Bv*RE, as well as the combination therapy exhibited marked protective effects against ifosfamide-induced nephro, hepato, neuro, and cardiotoxicity. Moreover, ifosfamide depicted a synergistic in vitro cytotoxic effect on HeLa cells in the presence of *Bv*RE. These results corroborate that the combination therapy of ifosfamide with *Bv*RE in cancer treatment can potentiate the anticancer effects of ifosfamide along with the amelioration of its conspicuous side effects.

## Introduction

Despite the rapidly growing knowledge base of human biology, cancer remains a daunting disease with numerous challenges ranging from diagnostic to treatment and responsible for around 13% of total mortalities globally^1^. Various chemotherapeutic agents alone or in combination are used widely for a variety of neoplastic diseases. Severe dose-dependent toxicities discern antineoplastic agents from other drugs as they cause a wide variety of multi-system toxicities^2^. Besides malignant tissues, many regenerating tissues (gastrointestinal tract mucosa, bone marrow, etc.) possess high proliferating ability and thus endure the toxic effects of chemotherapy^3^. These factors escalate the morbidity and mortality of cancer treatment.

Ifosfamide (3-(2-chloroethyl)-2-[(2-chloroethyl)amino]tetrahydro-2H1,3,2-oxazaphosphorine 2-oxide) is a nitrogen mustard and belongs to the oxazaphosphorine class. It is an alkylating agent with promising potentials in the treatment of various types of cancer, including lymphoma (Hodgkin and non-Hodgkin) and osteosarcoma, as well as, ovarian, testicular, soft tissue, lung, cervical, and breast cancer^4^. The mechanism of action of ifosfamide is not precisely known, and it is believed to act primarily through DNA alkylation resulting in cross-links mainly at guanine N-7 positions caused by isophosphoramide mustard, a metabolite of ifosfamide. The development of these irreversible intra and inter-strand cross-links in the DNA results in cell death^5^.

Besides the enormous therapeutic efficacy of ifosfamide, its safe usage is restricted due to the wide range of concurrent toxicities. Common toxicities encountered are hepatotoxicity, gastrointestinal abnormalities, nervous system toxicity, cardiotoxicity, local toxicity, hair follicle and skin toxicity, urinary tract toxicity, metabolic abnormalities, and gonadal toxicity^6^. Neurotoxicity is the most widely reported of all and occurs in almost 20% of patients with symptoms of severe hallucinations, confusion or somnolence seizures, and coma^7^. These side effects can range from acute to chronic, reversible to irreversible, trifling to potentially fatal, and their management is of extreme importance because they greatly influence the dose and course of treatment^8^. The maximum therapeutic benefits of ifosfamide requisite an antidote that can attenuate its induced toxicities with synergistic improvement in its efficacy as a chemotherapeutic agent.

Humanity is provided with an incredible treasure of remedies from nature in the form of enormous plant species. Since ancient times, medicinal plants have been widely used as therapeutic agents for several human ailments due to their versatile nature. Biologically active plant constituents have their proven role in attenuating the side effects of chemotherapeutic drugs. *Berberis vulgaris* (*B. vulgaris*) is a shrub in the family Berberidaceae, native to southern and central Europe, northwest Africa, and western Asia. It has an ethnopharmacologically rich history for the treatment of a large number of diseases^9^. In contrast to the historical pieces of evidence, various in vitro and in vivo studies have reported antioxidant, anticancer, antimicrobial, antipyretic, antidepressant, and antidiabetic potentials of *B. vulgaris*^9^*.* Phytochemical analyses of its extracts have revealed the presence of various bioactive constituents with preferably significant amounts of isoquinoline alkaloids (berberine, palmatine and jatrorrhizine) and bisbenzylisoquinoline alkaloids (oxyacanthine)^10^. The constituents of *B. vulgaris* are frequently used in the treatment of hepatitis, biliary fever, inflammation, gastrointestinal diseases, hemorrhages, gum inflammation, sore throat, malaria, diarrhea, and high blood cholesterol^11^. It is reported that the combined effects of phytochemicals with chemotherapeutic agents can enhance drug efficacy while reducing off-target effects^12^. *B. vulgaris* root and stem are primarily used for illnesses of vital organs such as liver, kidney, heart and circulatory system, gastrointestinal, and respiratory tract ailments^13,14^.

Berberine is the key bioactive ingredient of *B. vulgaris* with its highest abundance in roots. It is reported to possess potent hepato, nephro, and cardioprotective effects^9,15^. It could ameliorate these toxicities through its antioxidant, anti-inflammatory and antiapoptotic effects as well as via modulation of mitogen-activated protein kinase (MAPK) and nuclear factor-κB (NF-κB) signaling pathways^16^.

Multifactorial physiological activities of berberine, along with its antioxidant and anticancer activities, suggest that *B. vulgaris* root extract can potentially lower ifosfamide-induced toxicities and concurrently improve its activity as a chemotherapeutic agent. Considering reported potentialities of berberine and *B. vulgaris* root extract, the current study is based on in vivo experiments to evaluate the effects of orally administered methanol extract of *B. vulgaris* roots (*Bv*RE) in combination with ifosfamide against ifosfamide-induced nephro, hepato, cardio and neurotoxicity.

## Results

### Effect of *Bv*RE on ifosfamide-induced nephrotoxicity

Under different treatment conditions, kidney health was evaluated by examining serum albumin, creatinine, and blood urea nitrogen (BUN). Furthermore, MDA, GSH, and CAT levels of kidney tissues were determined to evaluate oxidative stress status (Table 1).

**Table 1:** Effect of *Bv*RE on ifosfamide-induced nephrotoxicity and oxidative stress on kidney tissue.

Groups	Serum albumin (g/dL)	Creatinine (mg/dL)	BUN (mg/dL)	MDA (μmol/mL)	GSH (nmol/mg)	CAT (U/mg protein)
Saline-treated control	4.6 ± 0.21	0.66 ± 0.16	19.8 ± 4.6	1.08 ± 0.2	0.36 ± 0.05	13.5 ± 1.4
Extract control A	4.8 ± 0.09^x^	0.78 ± 0.13^x^	23.8 ± 3.3^x^	1.5 ± 0.13^x^	0.32 ± 0.06^x^	15.59 ± 3.6^x^
Extract control B	4.6 ± 0.3^x^	0.83 ± 0.096^x^	21.3 ± 2.5^x^	0.97 ± 0.09^x^	0.27 ± 0.037^x^	13.6 ± 2.3^x^
Ifosfamide control A	3.1 ± 0.08^x^	2.65 ± 0.2^x^	79 ± 5.77^x^	1.27 ± 0.08^x^	0.26 ± 0.05^x^	10.8 ± 1.07^x^
Ifosfamide control B	3.5 ± 0.4^x^	2.41 ± 0.43^x^	71.8 ± 13^x^	1.4 ± 0.2^x^	0.25 ± 0.05^x^	10.42 ± 1.1^x^
Combination	3.68 ± 0.17^x,y,z^	0.98 ± 0.17^x,y,z^	22 ± 2.9^x,y,z^	1.04 ± 0.07^x,y,z^	0.32 ± 0.02^x,y,z^	14.6 ± 1.9^x,y,z^
Curative	3.48 ± 0.09^x,z^	1.18 ± 0.28^x,z^	23.3 ± 3.6^x,z^	1.42 ± 0.4^x,z^	0.33 ± 0.03^x,z^	13.6 ± 0.56^x,z^
Prophylactic	4.38 ± 0.48^x,y^	1 ± 0.19^x,y^	23.5 ± 2.9^x,y^	1.04 ± 0.09^x,y^	0.3 ± 0.05^x,y^	14.5 ± 4.5^x,y^

Descriptive statistics of renal function tests and oxidative stress parameters are represented as mean ± standard deviation of six observations (n = 6); where symbol x denotes a particular group compared with the saline-treated control as p < 0.05. Symbol y signifies the comparison of a particular group with plant controls as p < 0.05, while z expresses the comparison of a particular group with ifosfamide controls as p < 0.05 (one‐way ANOVA followed by post hoc Scheffe test).

The mean value of serum albumin in the saline-treated control group was 4.6 ± 0.21 g/dL. The ifosfamide control group A and ifosfamide control group B had the mean values of 3.1 ± 0.08 and 3.5 ± 0.4 g/dL, respectively, which were considerably lower than the saline control group. The decreased level of serum albumin in these groups is the marker of disturbed kidney function. A slight difference between both ifosfamide control groups (0.4 g/dL) reveals that the nephrotoxicity level was persistent even six days after the discontinuation of ifosfamide. The groups administered with only *Bv*RE, i.e., extract control A and extract control group B, the mean values of serum albumin (4.8 ± 0.09 and 4.6 ± 0.3 g/dL, respectively) were in agreement with the average value of the saline-treated control group. Serum albumin levels of saline and extract control groups have a statistically non-significant difference. In combination group (treated with both ifosfamide and *Bv*RE simultaneously) and curative group (treated with ifosfamide for 6 days with post-treatment of *Bv*RE for 6 days) the mean levels were 3.68 ± 0.17 and 3.48 ± 0.09 g/dL, respectively. These values indicate that serum albumin levels for combination and curative groups were increased compared to the ifosfamide treated groups and shifted slightly towards the saline-treated control group. Prophylactic group (pretreated with *Bv*RE for 6 days before the administration of ifosfamide for next 6 days) had an average value of 4.38 ± 0.48 g/dL for serum albumin which was close to the saline-treated control group, depicting the therapeutic effect of *Bv*RE against ifosfamide-induced nephrotoxicity in this pretreatment manner.

The mean value of creatinine was 0.66 ± 0.16 mg/dL for the saline-treated control group, and the mean value for ifosfamide control groups A and B were 2.65 ± 0.2 and 2.41 ± 0.43 mg/dL, respectively, presenting significant increase as compared to the saline-treated control group, illustrating considerable toxic effects on kidneys. Mean values for extract control groups A and B were analyzed as 0.78 ± 0.13 and 0.83 ± 0.096 mg/dL, respectively, close to the saline-treated control group. The mean for the combination group was calculated to be 0.98 ± 0.17 mg/dL that is lowered towards the average of the saline-treated control group, indicating a protective effect of *Bv*RE in revoking the renal functions towards normality. Similarly, the mean creatinine values for curative and prophylactic groups (1.18 ± 0.28 and 1 ± 0.19 mg/dL, respectively) were showed compensated renal function.

BUN for the saline-treated control group was recorded to be 19.8 ± 4.6 mg/dL. The mean value of BUN for ifosfamide control groups A and B were calculated as 79 ± 5.774 and 71.8 ± 13 mg/dL, respectively, significantly elevated compared to the saline-treated control group indicating disturbed renal functions. In extract control groups A and B, mean values for BUN (23.8 ± 3.3 and 21.3 ± 2.5 mg/dL, respectively) were closer to the saline-treated control group showing no noticeable difference in kidney functions. Mean values for combination, curative and prophylactic groups were calculated as 22 ± 2.9, 23.3 ± 3.6 and 23.5 ± 2.9 mg/dL, respectively, presenting slightly increased values but in a considerable agreement with the saline-treated control group, illustrating nephroprotective functions of *Bv*RE*.*

The value of the mean MDA level in the control group was observed to be 1.08 ± 0.2 μmol/mL. In ifosfamide control groups A and B, the mean values were increased to 1.27 ± 0.08 and 1.4 ± 0.2 μmol/mL, respectively. The same increase in the mean value of the MDA level was detected in extract control group A with a value of 1.5 ± 0.13 μmol/mL. In the plant control group B, it was evaluated to be 0.97 ± 0.09 μmol/mL. The mean value of the MDA level in combination and prophylactic groups was calculated to be 1.04 ± 0.07 and 1.04 ± 0.09 μmol/mL, respectively, showing very positive nephroprotective effects of *Bv*RE. The mean value for the MDA level in the curative group (1.42 ± 0.4 μmol/mL) was estimated close to the mean value of the control group specifying the defending effects of *Bv*RE.

GSH mean value of the saline-treated control group was observed as 0.36 ± 0.05 nmol/mg. Mean value of ifosfamide control group A (0.26 ± 0.05 nmol/mg) was lower than the mean of control group presenting the toxic effect of the drug, while after a 6-day gap (without administration of ifosfamide) the mean value of ifosfamide control group B was 0.25 ± 0.05 nmol/mg, indicating the persistent renal toxic effects of the drug. Mean values for extract-treated groups A and B were recorded to be 0.32 ± 0.06 and 0.27 ± 0.037 nmol/mg, which may be considered having no effects of plant extract on GSH levels. For combination, curative and prophylactic groups, the mean values were calculated as 0.32 ± 0.02, 0.33 ± 0.03 nmol/mg and 0.3 ± 0.05 nmol/mg, respectively, showing a marked difference from ifosfamide control groups and shift towards the saline-treated control group is a sign of protective effects of *Bv*RE.

**Figure 1:**
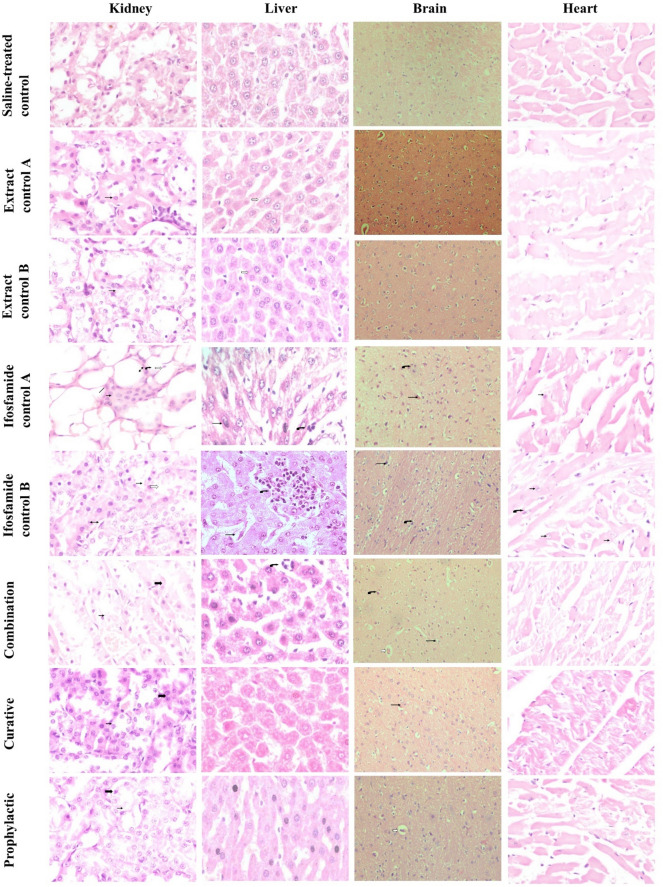
Histopathological findings (representative images) of kidney, liver, brain and heart across selected dose regimes of saline-treated control, extract control A, extract control B, ifosfamide control A, ifosfamide control B, combination, curative and prophylactic groups. Kidney: Nephrocyte destruction with dilation in Bowman’s space (curved arrow), epithelial desquamation (hollow arrow), inflammatory cells in glomeruli (two-sided arrow), flattening of the tubular epithelium (line), blood vessel congestion (arrow), and protein overload (blood arrow). Liver: Inflammatory cells infiltration (arrow), hepatocyte necrosis (curved arrow), and vacuole generation (hollow arrow). Brain: Demyelination and neuroinflammation (arrow), neuron degeneration (curved arrow), and dilated blood vessels (hollow arrow). Heart: Infiltrating inflammatory cells (arrow) and degeneration (curved arrow).

The mean value of CAT for the saline-treated control group was monitored as 13.5 ± 1.4 U/mg. In ifosfamide control groups A and B, the levels decreased to 10.8 ± 1.07 and 10.42 ± 1.1 U/mg, respectively, pointing the effect towards oxidative stress. In extract control group A, the CAT level was slightly raised to 15.59 ± 3.6 U/mg compared to the saline-treated group while extract control group B had nearly similar value as that of the saline-treated group (13.6 ± 2.3 U/mg). In the combination group, CAT concentration (14.6 ± 1.9 U/mg) was slightly higher than the saline-treated control group. For curative and prophylactic groups, the mean values were recorded to be 13.6 ± 0.56 and 14.5 ± 4.5 U/mg, respectively, implying protective effects of *Bv*RE administering periodically in pre and post-treatment manner. Histopathological renal findings (Table 2, Fig. 1) corroborated these results depicting the protective effects of *Bv*RE against ifosfamide-induced renal toxicity.

**Table 2:** Histopathological findings of *Bv*RE on ifosfamide-induced nephrotoxicity.

Group	Degeneration in tubular cells	Tubular cast	Glomerular congestion	Dilation in Bowman’s space	Epithelial desquamation	Blood vessel congestion	Inflammatory cells	Flattening of tubular epithelium	Protein overload
Saline-treated control	−	−	−	−	−	++	−	−	−
Extract control A	−	−	−	−	−	++	−	−	−
Extract control B	−	−	−	−	−	+++	−	−	−
Ifosfamide control A	−	−	−	+++	+++	+++	+ ^a^	+++	−
Ifosfamide control B	−	−	−	−	+++	++	+ ^b^	−	−
Combination	−	−	−	−	−	++	−	−	+++
Curative	−	−	−	−	−	++	−	−	+++
Prophylactic	−	−	−	−	−	++	−	−	+++

−  = not present, +  = slightly present, ++  = present, +++  = strongly present.

^a^In glomeruli.

^b^In perivascular.

The photomicrograph of the saline-treated control group showed normal kidney tissue architecture. Ifosfamide exposed groups A and B showed plentiful degenerations having a substantial dilation in Bowman’s space and epithelial desquamation. Slight presence of inflammatory cells was also observed in glomeruli and perivascular region in ifosfamide groups A and B. Flattening of the tubular epithelium was also observed in group A. Blood vessels were found to be congested in both. Histological examination of the rat kidneys in extract control groups A and B showed normal architecture like the saline-treated control group with blood vessel congestion. Analysis of combination, prophylactic and curative groups showed normal renal architecture compared to the saline-treated control group with the exception of protein overloaded.

### Effect of *Bv*RE on ifosfamide-induced hepatotoxicity

Serum ALT, AST, and ALP were selected as parameters for the evaluation of hepatotoxicity. Moreover, tissue MDA, GSH, and CAT levels were also measured as oxidative stress markers (Table 3). Findings of ifosfamide control groups showed a marked elevation in the levels of serum ALT, AST, ALP, tissue MDA, GSH, and CAT levels as compared to the saline-treated control group (p < 0.05), representing significant induction of hepatotoxicity. Results of combination, curative and prophylactic groups indicate that antidotal effects of *Bv*RE significantly restored the values of parameters towards the normal status in comparison to the control groups. Distinct therapeutic potential of plant extract was observed in prophylactic and combination therapy in comparison to the curative treatment (Table 3).

**Table 3:** Effect of *B. vulgaris* root extract (*Bv*RE) on ifosfamide-induced hepatotoxicity and oxidative stress on liver tissue.

Groups	ALT (U/L)	AST (U/L)	ALP (U/L)	MDA (µmol/mg)	GSH (nmol/mg)	CAT (U/mg)
Saline-treated control	42.24 ± 4.11	135.7 ± 9.42	115.7 ± 12.7	1.45 ± 0.45	0.36 ± 0.01	4.16 ± 1.24
Extract control A	43.25 ± 5.12^x^	145.25 ± 8.06^x^	127 ± 7.07^x^	1.361 ± 0.243^x^	0.40 ± 0.01	3.037 ± 1.948^x^
Extract control B	38.5 ± 6.66^x^	118.7 ± 9.91^x^	118.0 ± 12.52^x^	1.06 ± 0.03^x^	0.43 ± 0.03^x^	5.74 ± 1.11^x^
Ifosfamide control A	48.00 ± 6.9^x^	171.25 ± 9.25^x^	142.25 ± 4.3^x^	2.302 ± 0.285^x^	0.33 ± 0.03^x^	1.852 ± 0.385^x^
Ifosfamide control B	69.2 ± 6.39^x^	179.0 ± 11.16^x^	153.7 ± 9.29 ^x^	2.33 ± 0.79^x^	0.31 ± 0.04^x^	1.93 ± 0.28^x^
Combination	42.5 ± 5.69^x,y,z^	118.0 ± 4.32^x,y,z^	114.7 ± 9.64^x,y,z^	1.62 ± 0.68^x,y,z^	0.40 ± 0.03^x,y,z^	4.42 ± 3.9^x,y,z^
Curative	24.7 ± 4.79^x,z^	105.7 ± 5.06^x,z^	114.0 ± 7.53^x,z^	1.49 ± 0.42^x,z^	0.39 ± 0.08^x,z^	2.31 ± 0.22^x,z^
Prophylactic	33.2 ± 6.13^x,y^	93.5 ± 6.26^x,y^	102.5 ± 5.80^x,y^	1.73 ± 0.65^x,y^	0.44 ± 0.01^x,y^	3.04 ± 0.09^x,y^

Descriptive statistics of renal function tests and oxidative stress parameters are represented as mean ± standard deviation of six observations (n = 6); where symbol x denotes a particular group compared with the saline-treated control as p < 0.05. Symbol y signifies the comparison of a particular group with plant controls as p < 0.05, while z expresses the comparison of a particular group with ifosfamide controls as p < 0.05 (one‐way ANOVA followed by post hoc Scheffe test).

Liver tissue histopathology findings (Table 4, Fig. 1) affirmed ifosfamide-induced tissue damage as compared to normal control and tissue structure restoration potential of *Bv*RE against induced toxicity.

**Table 4:** Histopathological findings of *Bv*RE on ifosfamide-induced hepatotoxicity.

Group	Microvascular steatosis	Hepatocyte necrosis	Inflammation	Haemorrhage	Congestion
Saline-treated control	−	−	−	−	−
Extract control A	−	−	−	−	−
Extract control B	−	−	−	−	−
Ifosfamide control A	+++	+++	++	++	+++
Ifosfamide control B	+++	+++	+++	+++	++
Combination	−	+	+	−	−
Curative	+	−	+	−	−
Prophylactic	+	−	−	−	−

−  = not present, +  = slightly present, ++  = present, +++  = strongly present.

Ifosfamide resulted in a slight increase of ALT in ifosfamide control A (48.00 ± 6.9 U/L) as compared to the saline-treated control (42.24 ± 4.11 U/L). This effect was more pronounced in ifosfamide control B (69.2 ± 6.39 U/L) with around 1.6 times the elevation of ALT as compared to the saline-treated control group. On the other hand, there was no considerable change in ALT value in extract control group A (43.25 ± 5.12 U/L), while a slight decrease was observed in extract control B (38.5 ± 6.66 U/L). The groups treated with both ifosfamide and *Bv*RE in combination, curative and prophylactic manner presented ALT values as 42.5 ± 5.69, 24.7 ± 4.79, and 33.2 ± 6.13 U/L, respectively. So, combination treatment restored ALT value close to the saline-treated control while the rest of the two treatment schemes considerably reduced the value.

A reasonable elevation of AST was observed in ifosfamide control groups A and B (171.25 ± 9.25 and 179.0 ± 11.16 U/L, respectively) as compared to the saline-treated control group (135.7 ± 9.42 U/L). While, extract control groups A and B presented AST values as 145.25 ± 8.06 and 118.7 ± 9.91 U/L, respectively. A depletion in AST levels was observed in combination, curative and prophylactic groups with 118.0 ± 4.32, 105.7 ± 5.06, and 93.5 ± 6.26 U/L, respectively.

A relatively different trend was observed in ALP with 115.7 ± 12.7 U/L in the saline-treated control and increased levels to 142.25 ± 4.3 and 153.7 ± 9.29 U/L in ifosfamide control A and B, respectively. A very slight ALP increase was seen in extract control group A (127 ± 7.07 U/L) and extract control group B (118.0 ± 12.52 U/L). In combination and curative groups (114.7 ± 9.64, and 114.0 ± 7.53 U/L, respectively), it was found well close to the saline-treated control while a slight decrease to 102.5 ± 5.80 U/L was depicted in prophylactic group.

Among tissue oxidative stress parameters, MDA levels were considerably increased in ifosfamide control groups, with around 60% increase in both the groups as compared to the saline-treated control. The rest of the groups showed values close to the saline-treated control group except around a 26% decrease in extract control group B and a 19% increase in the prophylactic group. GSH level was not significantly affected among different groups as compared to the saline-treated control group (0.36 ± 0.01 nmol/mg). Although, a slight decrease was seen in ifosfamide control groups while a minor increase was observed in the rest of the groups as compared to the saline-treated control group.

Administration of ifosfamide significantly decreased CAT concentration in liver tissues with more than fifty percent drop in ifosfamide control groups A and B (1.852 ± 0.385 and 1.93 ± 0.28 U/mg, respectively) as compared to the saline-treated control (4.16 ± 1.24 U/mg). On the other hand, *Bv*RE induced a decrease in CAT value in extract control group A (3.037 ± 1.948 U/mg) and an increase in extract control group B (5.74 ± 1.11 U/mg). The combination course of treatment proved most promising in restoring CAT value (4.42 ± 3.9 U/mg) close to the saline-treated control. In curative and prophylactic treatments, the values (2.31 ± 0.22 and 3.04 ± 0.09 U/mg, respectively) were less than saline-treated control but greater than ifosfamide control groups.

Histologic analysis of the saline-treated control group rats’ liver revealed the normal architecture of hepatocytes. The structure of the ifosfamide control groups was significantly affected by ifosfamide administration presenting ifosfamide-induced toxicity. The destruction of hepatocytes with the disarray of hepatocyte architecture was observed along with hepatocyte necrosis (arrow). Fatty changes, microvascular steatosis, and infiltration of inflammatory cells in portal areas (curved arrow) were also seen. Histologic examination of the rat liver in extract control group A showed normal architecture as of control, while slight fatty changes and vacuole generation (arrows) were observed in plant control B. The combination group revealed slight hepatocyte swelling and necrosis (arrow). Inflammatory cell infiltration was also present along with vacuole generation. The prophylactic group revealed the initiation of significant regenerative changes. More regular alignment of hepatocytes was observed in the prophylactic group while the curative group depicted mild hepatocyte swelling, fatty degeneration, and microvascular steatosis.

### Effect of *Bv*RE on ifosfamide-induced neurotoxicity

AChE and oxidative stress markers (MDA, GSH and CAT) were selected to examine the *bona fide* encephalopathy of ifosfamide, and the results are presented in Table 5. Findings revealed a significant decrease in brain tissue GSH, AChE, and CAT levels as well as the increase in tissue MDA level in ifosfamide control groups A and B in comparison to the saline-treated control group, indicating ifosfamide-induced neurotoxicity. While statistical figures of groups treated with *Bv*RE in all three regimens (combination, curative and prophylactic) depicted the potential of *B. vulgaris* as an antidote against ifosfamide-induced neurotoxicity.

**Table 5:** Effect of *Bv*RE on ifosfamide-induced neurotoxicity and oxidative stress on brain tissue.

Groups	AChE (µmol/g)	MDA (µmol/mg)	CAT (U/mg)	GSH (nmol/mg)
Saline-treated control	74.0 ± 10.4	0.91 ± 0.025	1.92 ± 0.05	0.12 ± 0.02
Extract control A	82.29 ± 18.0^x^	0.905 ± 0.062^x^	1.89 ± 0.08^x^	0.090 ± 0.012^x^
Extract control B	93.2 ± 6.6^x^	0.94 ± 0.13^x^	1.91 ± 0.09^x^	0.10 ± 0.01^x^
Ifosfamide control A	63.69 ± 12.92^x^	1.791 ± 0.476^x^	1.654 ± 0.061^x^	0.104 ± 0.014^x^
Ifosfamide control B	66.0 ± 9.9^x^	1.87 ± 0.61^x^	1.61 ± 0.06^x^	0.08 ± 0.01^x^
Combination	83.1 ± 11.6^x,y,z^	0.85 ± 0.01^x,y,z^	2.01 ± 0.12^x,y,z^	0.09 ± 0.003^x,y,z^
Curative	71.7 ± 13.1^x,z^	0.99 ± 0.18^x,z^	1.81 ± 0.11^x,z^	0.12 ± 0.006^x,z^
Prophylactic	92.3 ± 23.1^x,y^	0.92 ± 0.08^x,y^	1.99 ± 0.15^x,y^	0.12 ± 0.01^x,y^

Descriptive statistics of renal function tests and oxidative stress parameters are represented as mean ± standard deviation of six observations (n = 6); where symbol x denotes a particular group compared with the saline-treated control as p < 0.05. Symbol y signifies the comparison of a particular group with plant controls as p < 0.05, while z expresses the comparison of a particular group with ifosfamide controls as p < 0.05 (one‐way ANOVA followed by post hoc Scheffe test).

Histological studies support the therapeutic potential of *Bv*RE as well (Fig. 1, Table 6). The ameliorative potential of the plant extract is more pronounced in trails of the pretreatment category as compared to post-treatment and combination therapy groups.

**Table 6:** Histopathological findings of *Bv*RE on ifosfamide-induced neurotoxicity.

Group	Hippocampal neurons	Necrotic changes	Demyelination	Inflammation
Saline-treated control	−	−	−	−
Extract control A	−	−	−	−
Extract control B	−	−	−	−
Ifosfamide control A	+++	+++	+++	+++
Ifosfamide control B	+++	++	++	+++
Combination	−	-	+	+
Curative	+	+	++	++
Prophylactic	+	+	+	+

−  = not present, +  = slightly present, ++  = present, +++  = strongly present.

The photomicrograph of the saline-treated control group showed normal neurons with prominent nuclei in a fibrillary background, and similar fashion was observed in groups treated with *Bv*RE. Ifosfamide exposed groups A and B showed numerous degenerating neurons that appeared shrunken and dark. In addition, demyelination and neuroinflammation were also observed along with dilated blood vessels. The analysis of the curative group showed some degenerating neurons, demyelination and dilated blood vessels along with mild neuroinflammation. Histologic analysis of the prophylactic group revealed normal neurons in a fine fibrillary background with only a few dilated blood vessels revealing the initiation of significant regenerative changes. The examination of the combination group showed mild degeneration, dilated blood vessels, and minimal neuroinflammation was present.

### Effect of *Bv*RE on ifosfamide-induced cardiotoxicity

The results of heart tissue oxidative stress and blood LDH are presented in Table 7.

**Table 7:** Effect of *Bv*RE on ifosfamide-induced cardiotoxicity and oxidative stress on heart tissue.

Groups	LDH (mmol/mg)	MDA (µmol/mg)	GSH (nmol/mg)	CAT (µmol/mg)
Saline-treated control	1.545 ± 0.085	1.1 ± 0.024	0.044 ± 0.01	5.3 ± 0.28
Extract control A	1.347 ± 0.298^x^	0.9 ± 0.04^x^	0.04 ± 0.008^x^	4.7 ± 0.12^x^
Extract control B	1.199 ± 0.095^x^	0.97 ± 0.03^x^	0.038 ± 0.01^x^	5.6 ± 0.53^x^
Ifosfamide control A	3.456 ± 0.264^x^	1.36 ± 0.08^x^	0.022 ± 0.01^x^	5.11 ± 0.35^x^
Ifosfamide control B	1.823 ± 0.608^x^	1.63 ± 0.15^x^	0.018 ± 0.009^x^	5.4 ± 0.35^x^
Combination	1.635 ± 0.24^x,y,z^	1.18 ± 0.2^x,y,z^	0.02 ± 0.01^x,y,z^	5.73 ± 0.72^x,y,z^
Curative	1.339 ± 0.097^x,z^	1.3 ± 0.3^x,z^	0.04 ± 0.01^x,z^	5.36 ± 0.58^x,z^
Prophylactic	0.807 ± 0.165^x,y^	1.01 ± 0.07^x,y^	0.032 ± 0.01^x,y^	5.44 ± 1.61^x,y^

Descriptive statistics of renal function tests and oxidative stress parameters are represented as mean ± standard deviation of six observations (n = 6); where symbol x denotes a particular group compared with the saline-treated control as p < 0.05. Symbol y signifies the comparison of a particular group with plant controls as p < 0.05, while z expresses the comparison of a particular group with ifosfamide controls as p < 0.05 (one‐way ANOVA followed by post hoc Scheffe test).

For the saline-treated control, the mean value for the LDH level was observed to be 1.545 ± 0.085 mmol/mg. Mean values in ifosfamide control groups A and B were obtained as 3.456 ± 0.264 and 1.823 ± 0.608 mmol/mg, respectively, depicting increased LDH levels during the ifosfamide administration and marked restoration after 6 days of the suspension of ifosfamide administration. The extract control groups A and B presented a slight decrease to 1.347 ± 0.298 and 1.199 ± 0.095 mmol/mg, respectively, as compared to the saline-treated control group. The mean LDH in the combination group (1.635 ± 0.24 mmol/mg) found slightly increased as compared to the saline-treated control group. Curative and prophylactic groups presented decreased LDH levels of 1.339 ± 0.978 and 0.807 ± 0.165 mmol/mg, respectively, compared to the saline-treated control group.

The value of the mean MDA level in the control group was observed to be 1.1 ± 0.024 µmol/mg. Mean value in ifosfamide control group A was increased to 1.36 ± 0.08 µmol/mg, which was further elevated in ifosfamide control group B (1.63 ± 0.15 µmol/mg). In extract control group A, a decrease in the mean value of the MDA level was detected with a value of 0.9 ± 0.04 µmol/mg. In extract control group B it was evaluated to be 0.97 ± 0.03 µmol/mg. The mean value of MDA level in combination and prophylactic groups were calculated (1.18 ± 0.2 and 1.01 ± 0.07 µmol/mg, respectively) were relatively closer to the saline-treated control group while in the curative group no significant recovery was observed with a value of 1.3 ± 0.3 µmol/mg, specifying the positive effects of *Bv*RE only in combination and prophylactic regimes.

Similarly, the mean GSH mean values of ifosfamide control groups A and B (0.022 ± 0.01 and 0.018 ± 0.009 nmol/mg, respectively) were significantly lowered as compared to the saline-treated control group (0.044 ± 0.01 nmol/mg). This indicates persistent oxidative stress even 6 days after the termination of ifosfamide administration. Mean values for extract control groups A and B were recorded to be 0.04 ± 0.008 and 0.038 ± 0.01 nmol/mg, which indicates no significant effects of *Bv*RE on GSH levels. For combination, curative and prophylactic groups, the mean values were observed as 0.02 ± 0.01, 0.04 ± 0.01 and 0.032 ± 0.03 nmol/mg, respectively, showing a marked recovery in GSH levels in curative and prophylactic treatments while no effect in combined administration of *Bv*RE and ifosfamide.

The mean value of CAT for the saline-treated control group was monitored as 5.3 ± 0.28 µmol/mg. Overall, no significant difference in CAT levels was observed in cardiac tissues of different groups, showing sustained redox homeostasis during selected treatments.

Histopathological findings of cardiac tissues were in agreement with the biochemical outcomes, depicting the protective effects of *Bv*RE against ifosfamide-induced cardiac toxicity. The results are presented in Fig. 1 and Table 8.

**Table 8:** Histopathological findings in the heart.

Group	Myocardial cell swelling	Degeneration	Loss of transverse striations	Infiltrating inflammatory cells
Saline-treated control	−	−	−	−
Extract control A	−	−	−	−
Extract control B	−	−	−	−
Ifosfamide control A	−	−	−	+++
Ifosfamide control B	−	+++	−	+++
Combination	−	−	−	−
Curative	−	−	−	−
Prophylactic	−	−	−	−

−  = not present, +  = slightly present, ++  = present, +++  = strongly present.

The photomicrograph of the saline-treated control group showed normal structure. Ifosfamide exposed groups A and B showed infiltrating inflammatory cells while degeneration was observed in ifosfamide control group B. Extract control groups A and B showed normal architecture like the saline-treated control group without any degeneration. Analysis of combination, curative and prophylactic groups showed normal architecture (same as the saline-treated control group); hence, *Bv*RE at 1000 mg/kg displayed marked protection against cardiotoxicity induced by ifosfamide.

### Effect of *Bv*RE and ifosfamide on blood count, cholesterol, and triglycerides

The effect of different dose regimes on hemoglobin, cholesterol, and triglyceride levels and white blood cell, red blood cell, platelet, neutrophils, and lymphocyte counts are given in Table 9.

**Table 9:** Blood count (Hb, WBCs, RBCs, PLTs, Nue, and Lym) cholesterol and triglyceride levels for selected groups.

Groups	Hb (g/dL)	WBCs (10^3^/µL)	RBCs (10^6^/µL)	PLTs (10^3^/mL)	Neu (10^3^/µL)	Lym (10^3^/µL)	Cholesterol (mg/dL)	Triglyceride (mg/dL)
Saline-treated control	13.2 ± 1.36	9.73 ± 1.9	7.85 ± 1.04	398.2 ± 55.6	2.7 ± 0.47	7.3 ± 1.11	84.8 ± 10.8	59.8 ± 2.2
Extract control A	13.2 ± 0.9	8.35 ± 1.2	7.28 ± 1.19	455 ± 63.16	2.03 ± 0.7	7.05 ± 0.64	73.8 ± 6.5	60 ± 6.98
Extract control B	13.6 ± 0.4	6.93 ± 0.57	7.9 ± 0.7	432.5 ± 72	2.25 ± 0.94	7.25 ± 1.04	64.5 ± 5.06	50.3 ± 6.3
Ifosfamide control A	12.9 ± 0.94	5.0 ± 0.93	5.8 ± 0.82	73 ± 28.25	4.35 ± 1.01	3.15 ± 0.76	92.5 ± 6.6	86.3 ± 5.38
Ifosfamide control B	12.4 ± 0.9	5.45 ± 0.74	4.12 ± 0.31	91.25 ± 13.12	3.95 ± 0.99	3.45 ± 0.69	88 ± 6.8	79.5 ± 6.03
Combination	11.8 ± 1.07	9.48 ± 2.2	7.38 ± 1.21	313.75 ± 96.2	3.0 ± 0.9	6.25 ± 1.8	81.3 ± 4.65	70.5 ± 10.25
Curative	12.8 ± 1.5	8.88 ± 0.92	6.45 ± 0.6	320.5 ± 113.7	3.2 ± 1.6	6.5 ± 1.9	52.5 ± 6.8	45.5 ± 1.3
Prophylactic	12.5 ± 0.4	9.025 ± 2.1	6.6 ± 1.78	345 ± 51.56	2.9 ± 0.36	6.98 ± 1.5	58.75 ± 2.87	60.25 ± 8.38

*Hb* haemoglobin, *WBCs* white blood cells, *RBCs* red blood cells, *PLTs* platelets, *Neu* neutrophils, *Lym* lymphocytes.

The values are represented as mean ± standard deviation (mean ± SD) of six values (n = 6).

The mean value of hemoglobin in saline-treated control was recorded as 13.2 ± 1.36 g/dL, and there was no significant variation across the different groups. Among different treatment groups, the white blood cell count represented considerable depression in both extract and ifosfamide control groups as compared to the control group. Comparison of the mean value of the saline-treated control group (9.73 ± 1.9 × 10^3^/µL) with plant extract control groups A and B (8.35 ± 1.2 and 6.93 ± 0.57 × 10^3^/µL, respectively) revealed a marked decrease in white blood cell count. On the other hand, mean values among ifosfamide control groups A and B (5.0 ± 0.93 and 5.45 ± 0.74 × 10^3^/µL, respectively) also represents a marked decrease in white blood cell count. However, noticeable restoration effects of *Bv*RE has been observed upon all the treatment groups including combination group (9.48 ± 2.2 × 10^3^/µL), curative group (8.88 ± 0.92 × 10^3^/µL) and prophylactic group (9.025 ± 2.1 × 10^3^/µL) in comparison to mean value of control group. The current results scenario is debatable and needs future investigation about the effect of *B. vulgaris* individually and in combination with ifosfamide on white blood cells count.

Saline-treated control represented the mean value of red blood cells as 7.85 ± 1.04 10^6^/mm^3^ while in comparison plant control groups A and B have the mean values of 7.28 ± 1.19 and 7.9 ± 0.7 × 10^6^/µL, respectively, representing no significant difference among normal control and both plant control groups. Conversely, a noticeable decrease has been observed in RBCs count of ifosfamide treated control groups A and B (5.8 ± 0.82 and 4.12 ± 0.31 × 10^6^/µL) in comparison to the saline-treated control group, demonstrating that ifosfamide treatment negatively affects the normal status of the red blood cell count. However, in comparison to control group, mean values of treatment groups, i.e., combination group (7.38 ± 1.21 × 10^6^/µL), curative group (6.45 ± 0.6 × 10^6^/µL) and prophylactic group (6.6 ± 1.78 × 10^6^/µL) depicted that plant extract effectively revert the RBCs level towards normal in comparison to the normal control group.

The mean value of platelets from saline-treated normal control is 398.2 ± 55.6 × 10^3^/mL, whereas in comparison plant control groups A and B have the mean value as (455 ± 63.16 and 432.5 ± 72 × 10^3^/mL, respectively), which represents no significant difference among normal control and both extract control groups. Conversely, a noticeable decrease has been observed in platelet count of ifosfamide treated control groups A and B (73 ± 28.25 and 91.25 ± 13.12 × 10^3^/mL) in comparison to the saline-treated control group, demonstrating that ifosfamide treatment negatively affects the normal status of platelets. On the other hand, in comparison to control group, mean values of treatment groups, i.e., combination group (313.75 ± 96.2 × 10^6^/mm^3^), curative group (320.5 ± 113.7 × 10^6^/µL) and prophylactic group (345 ± 51.56 × 10^6^/µL) depicted restoration effects of plant extract which turns the platelets count towards normal as compared to control group.

The mean value of neutrophils noted as 2.7 ± 0.47 × 10^3^/µL while negative control groups A and B have the mean value 4.35 ± 1.01 and 3.95 ± 0.99 × 10^3^/µL, respectively, which is noticeably higher than that of the saline-treated control group showing that ifosfamide treatment affects the normal levels of neutrophils. The groups administered with plant extract had mean values of neutrophils nearly close to the saline-treated control group. However, in comparison to control groups, the mean values of treatment groups, i.e., combination (3.2 ± 0.9 × 10^3^/µL), curative (3.2 ± 1.6 × 10^3^/µL) and prophylactic group (2.9 ± 0.36 × 10^3^/µL) depicted that plant extract in combination with ifosfamide, as post- and pretreatment agent effectively reverts the neutrophils level towards normal.

The mean value of lymphocytes observed as 7.3 ± 1.11 × 10^3^/µL while ifosfamide control groups A and B have the mean values of 3.15 ± 0.76 and 3.45 ± 0.69 × 10^3^/µL, respectively, which is considerably lower than that of the saline-treated control group showing that ifosfamide treatment negatively affects the normal lymphocytes status. The groups administered with plant extract (extract control groups A and B 7.05 ± 0.64 and 7.25 ± 1.04 × 10^3^/µL, respectively) has no significant difference as compared to the saline-treated control group. Though in comparison to ifosfamide control groups, mean values of treatment groups (combination 6.25 ± 1.8 × 10^3^/µL, curative 6.5 ± 1.9 × 10^3^/µL, and prophylactic group 6.98 ± 1.5 × 10^3^/µL) that plant extract has a marked effect on the restoration of the normal status of lymphocytes.

The mean value of total cholesterol in the saline-treated control group was found at 84.75 ± 10.8 mg/dL. For ifosfamide control groups A and B, the mean values were 92.5 ± 6.61 and 88 ± 6.83 mg/dL, respectively, which is considerably higher than normal value. On the other hand, extract control groups A and B presented mean values of 73.75 ± 6.50 and 64.5 ± 5.06 mg/dL, respectively, with depression as compared to the saline-treated group. In the combination group, the simultaneous treatment of ifosfamide and *Bv*RE slightly lowered total cholesterol level (81.25 ± 4.65 mg/dL) as compared to the saline-treated control while in curative and prophylactic groups average value of total cholesterol was 52.5 ± 6.76 and 58.75 ± 2.87 mg/dL, respectively, representing evident restoration effects of plant extract on equilibration of the total cholesterol level towards normal status.

The mean value of serum triglycerides in the saline-treated control group was observed as 59.75 ± 2.2 mg/dL. The ifosfamide control groups A and B had considerably higher values of 86.25 ± 5.37 and 79.5 ± 6.03 mg/dL, respectively. In extract control groups A and B serum triglyceride levels were 60 ± 6.97 and 50.25 ± 6.29 mg/dL, respectively. In the combination group, the mean serum triglyceride value (70.5 ± 10.25 mg/dL) was higher than the saline-treated control but lower than the ifosfamide control groups. In curative and prophylactic groups, the mean values of serum triglycerides were 45.5 ± 1.29 and 60.25 ± 8.38 mg/dL, respectively, representing positive restoration effects of *Bv*RE on ifosfamide-induced dyslipidemia.

### In vitro effect of *Bv*RE, ifosfamide, and combination on HeLa cells

**Figure 2:**
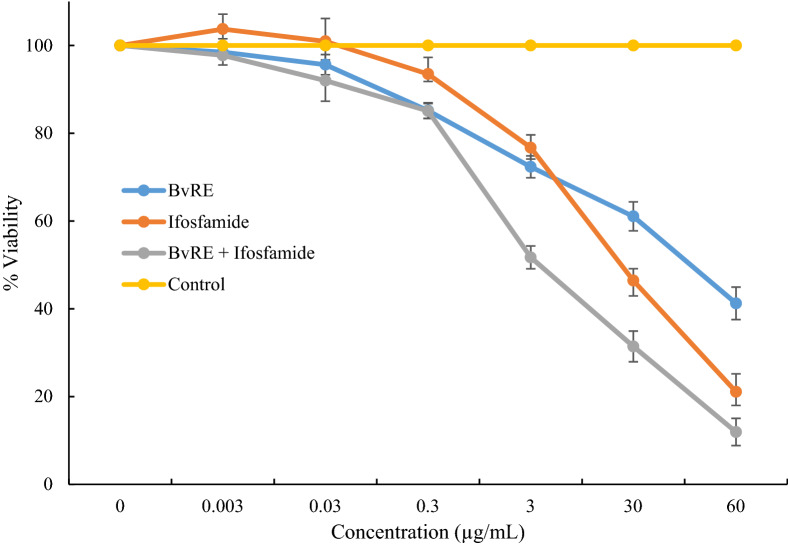
Dose–response curve of *Bv*RE, ifosfamide, and combination against HeLa cells using MTT assay.

MTT assay was performed to study the combined effect of *Bv*RE on cytotoxicity of ifosfamide using HeLa cells. The cells were treated with different concentrations (0.003, 0.03, 0.3, 30, and 60 µg/mL) of *Bv*RE, ifosfamide, and their combination, and percentage viabilities were calculated to plot dose–response curves (Fig. 2). At a test concentration of 30 µg/mL, the percentage cell viabilities for *Bv*RE, ifosfamide, and combination were 61, 46.4, and 31.4%, respectively. The *IC*_*50*_ values of *Bv*RE, ifosfamide, and combination were 49.7 ± 3.65, 26.82 ± 3.8, and 3.14 ± 0.21 µg/mL, respectively.

The combination index of *Bv*RE and ifosfamide at *IC*_*50*_ was (3.14/49.7) + (3.14 + 26.82) = 0.18. The combination index value considerably lower than 1 indicates the significant synergistic potential of *Bv*RE and ifosfamide combination on cytotoxicity.

## Discussion

Ifosfamide has been stated as a potent and efficient anticancer agent, used as a front-line treatment in various types of cancers^17^. It has a moderate half-life of 7 h, but its dose-dependent toxicities mainly limit its therapeutic effects^18^. It is a prodrug, metabolized by cytochrome P450 to its active alkylating agents, 4-hydroxy-ifosfamide, and isofosfamide mustard^19^ along with other non-alkylating metabolites mainly chloroacetaldehyde and acrolein, which may be responsible for the toxicities of ifosfamide, mainly, neuro and nephrotoxicity and moderate cardio and hepatotoxicity^20^.

For the treatment of various toxicities, counteractive effects of herbs have been famed from the many prehistoric times. Plants are achieving the role of major health care reserves worldwide and the potential of herbs and plant-based remedies have progressively been known for their precautionary action and the treatment of human diseases. Almost one-fourth of recommended drugs are made from the extracts of plants or their effective components^21^.

*Bv*RE root extract possesses antimicrobial, antidiabetic, and antitumor properties as well as is known for its nephroprotective, hepatoprotective, and lipid-lowering properties^22^. Experimental and clinical evidence suggests that it is beneficial in combating nervous system toxicity and hepatic toxicity induced by anticancer drugs^23^. Berberine is a primary constituent of *Bv*RE, mainly responsible for its protective role, so, we have previously reported HPLC-based quantification of berberine in *Bv*RE^15^. The same extract having 10.29% berberine was used in this study. The *LD*_*50*_ of berberine has been observed higher than 1000 mg/kg in rats^24^. Hence, while selecting the dose of *Bv*RE, the amount of extract delivering 100 mg of active berberine (1000 mg *Bv*RE) was selected, which was considered a safe therapeutic dose.

The current study demonstrates that *Bv*RE could avoid different toxicities associated with ifosfamide administration in an animal model. Ifosfamide's various dose levels ranging between 40 to 80 mg/kg have been used to illustrate toxic effects in rats^25,26^. Here, a clinically appropriate dosage of ifosfamide (50 mg/kg/days, *i.p.*, 6 days) was selected, which resulted in distorted renal, hepatic, neural and cardiac functions, as shown by the respective biomarkers, tissue oxidative stress parameters, and histopathology. *Bv*RE (1000 mg/kg/day, orally, 6 days) with ifosfamide in selected dose regimes produced significant suppression of toxicities associated with ifosfamide administration. Additionally, the administration of *Bv*RE effectively prevented ifosfamide-induced bodyweight reduction and mortality in rats. This finding recommends that *Bv*RE may have a shielding role in ifosfamide-induced toxicities and can be anticipated as a possible agent for reducing the severe complications of ifosfamide in clinical practice.

In the present study, a comparison of combination, curative, and prophylactic dose regimes were evaluated for their effects on ifosfamide-induced toxicities. Ifosfamide's administration resulted in an increase in MDA level and decrease in CAT and GSH activity compared to the saline-treated group in all four tested organs. This indicates a possible mechanism behind the ifosfamide-induced toxicities. The difference was relatively pronounced in the liver and brain, a representation of the *bona fide* ifosfamide toxicity profile. The reduction in intracellular GSH levels was noticeable at the initial stages of toxicity induced by ifosfamide. This reduction of tissue GSH is among key factors responsible for lipid peroxidation in cell membranes, distressing the functional integrity of the cellular structure, and if the damage is severed, cell death is unavoidable^27^. The rise of the MDA level described by the current study confirms the above mechanism. As the crucial cellular defense mechanism in the form of GSH weakens, cells are more vulnerable to the oxidative stress triggered by the metabolites such as chloroacetaldehyde. In parallel, the CAT level reduction is an exact representation of oxidative stress.

*Bv*RE has strong antioxidant acidity due to the abundance of isoquinoline alkaloids in it^10^ which can act via two major protective mechanisms against ifosfamide toxicity, to act as a precursor for GSH synthesis, as well as, a free radical and oxidant scavenger against reactive oxygen species via conjugation with reactive metabolites and converting into relatively safer compounds. Hence it is an ultimate aspirant for restocking tissue GSH levels^28^.

Renal toxicity is demonstrated by most of the chemotherapeutic agents and serves as a major dose-limiting side effect. It is manifested by the decreased glomerular filtration rate resulting in increased serum creatinine and blood urea nitrogen and reduced serum albumin. Elevation of these analytes in the serum of ifosfamide treated rats referred to the renal cellular injury^29^ as damaged renal cells develop leaky membranes, permitting the escape of these components into the bloodstream, raising their levels in the serum^30^.

The ifosfamide-induced renal impairment is a direct result of tubular damage leading ultimately to renal failure. The underlying mechanism of this effect is not well-understood^31^. It has been established that ifosfamide and its metabolites are preferentially taken up and accumulated in nephrocytes, resulting in an enhanced production of ROS leading to oxidative stress that could be the underlying cause of adverse effects on kidneys. One the other hand, acrolein is considered responsible for hemorrhagic cystitis, which can be addressed by administering a thiol compound mesna. This co-administration has been found ineffective against nephrotoxicity^32^. Numerous studies have reported a possible role of antioxidants in protecting kidneys against ifosfamide-induced toxicity^33^. The investigations have revealed that *B. vulgaris* constitute several therapeutic components with potent antioxidant effects, e.g., berberine^34^.

Furthermore, orally administered berberine has a *T*_max_ of 2.6 h with *C*_max_ of 9.48 ng/mL^35^. Berberine and its metabolites are preferentially accumulated in the kidney^36^, which results in the induction of diuresis via saluretic effect^37^. These properties and the administration of its high dose (1000 mg/kg) explain the elevated MDA levels in extract control group A, which dropped down to the normal value in extract control groups B. Similar behavior has already been observed in our previous study^15^.

It has been found that ifosfamide is accumulated and metabolized in hepatocytes through cytochrome P450 producing active 4-hydroxyifosfamide, inactive 3-dechloroethylifosfamide or 2-dechloroethylifosfamide, and toxic acrolein and chloroacetaldehyde. These metabolites trigger an enhanced production of reactive oxygen species (ROS), which give rise to oxidative stress^5,32^. The oxidative stress, in turn, initiates lipid peroxidation, decreased antioxidant defense, protein and DNA damage, and ultimately cell damage resulting in altered parameters of liver function.

Neurotoxicity is among the most challenging factor of ifosfamide treatments. Ifosfamide-induced encephalopathy could manifest mild symptoms of fatigue to serious outcomes leading to death^38^. The mechanism involves the interaction of ifosfamide with cytochrome CYP3A4/2B6 resulting in the formation of chloroacetaldehyde, a potentially neurotoxic compound. One the other hand, berberine has been reported to strongly inhibit CYP3A4^39,40^. From these observations, it could be hypothesized that combination or post-treatment of *Bv*RE can lower the production of chloroacetaldehyde and thus protecting against encephalopathy. The results in the present study have demonstrated visible encephalopathy in ifosfamide treated groups while *Bv*RE has significantly lowered this effect, particularly in prophylactic and combination groups.

Cardiotoxicity is a typical onset of chemotherapy that comprises various effects ranging from minor blood pressure changes and arrhythmias to cardiomyopathy. In literature, various chemotherapy-induced cardiotoxicity mechanisms are suggested, including cellular damage due to the formation of ROS and the induction of immunogenic reactions with antigen-presenting cells in the heart^41^. Furthermore, the effect of the anticancer drugs on some phospholipids, particularly cardiolipin could be responsible for cardiotoxicity. A sparse amount of literature is available underlying the effects of ifosfamide on the cardiovascular system. It is reported that its high dose usually induces some reversible effects, including malignant arrhythmias and myocardial depression^42^. As revealed by the current investigation, ifosfamide caused cardiotoxicity, which included the higher levels of lactate dehydrogenase compared with the control. Oxidative stress parameters (MDA, CAT and GSH) have also indicated disturbed redox homeostasis in the heart tissues, which was efficiently restored by *Bv*RE.

The increase in lipid profile parameters was more evident in ifosfamide control A (dissected after 6 days) while the values were reverted towards the saline-treated control group in ifosfamide control B (dissected after 12 days), which could be due to the response of innate recovery mechanism after the stress period. It is evident from the results that ifosfamide toxicity affects the normal status of lipid profile while administration of *B. vulgaris* in three tested treatment groups showed restoration of imbalances towards the normal range.

A considerable decrease in HeLa cell viability was observed with combined treatment at concentrations higher than 0.3 µg/mL. This evidence validates the synergistic cytotoxic effect of *Bv*RE in combination with ifosfamide against selected cancer cells^32^. Ifosfamide, being DNA cross-liner produces irreversible links between DNA strands. Berberine in *Bv*RE has also been reported to interact with DNA inducing cell cycle arrest in various cancer cells^43^. This analogy in the mode of action could be responsible for the synergistic cytotoxicity of the combination against HeLa cells. Moreover, berberine can also interact with various proteins involved in cell growth, cell cycle, metastasis, and angiogenesis^44^. These interactions could probably block cell survival pathways initiated due to ifosfamide resulting in an increased sensitivity and decreased viability.

## Methods

### Animals

Animals for the experiment were male albino rats of 200–300 g. Animals were provided with optimized conditions throughout the experiment, including standard cages, standard rat food and free water access, and 12 h light and dark period along with controlled temperature 22 ± 2 °C. All procedures and protocols of experiments were endorsed by the local Ethics Committee of the University of Lahore (approval number “IMBB-16-0035”) under the regulations of the National Institute of Health (NIH, USA) guide for the care and use of laboratory animals.

### Chemicals

Ifosfamide (Pharmedic, Pakistan), berberine chloride (Alfa Aesar, Germany), and remaining analytical grade chemicals (Sigma Aldrich, Germany) used in this study were purchased from the commercial sources. The biochemical tests were performed using kits (Human Diagnostics, Germany).

### Preparation of *Bv*RE and quantification of berberine

Information regarding plant collection, taxonomy, preparation of the *Bv*RE, and quantification of berberine in the extract using HPLC has already been reported in our previous account^15^.

### Treatment protocol

Rats were randomly divided into eight groups of 6 rats in each (n = 6). The total experimental duration was of 12 days, followed by dissection on the 13^th^ day. Ifosfamide (50 mg/kg of body weight) and *Bv*RE (1000 mg/kg of body weight) were prepared in injection water and injected via intraperitoneal and oral routes, respectively. The treatment protocol includes one control and seven trial groups as presented in Table 10.

**Table 10:** Rat model in vivo dose regime and treatment protocol to study the protective effects of *Berberis vulgaris* root extract (*Bv*RE) on ifosfamide-induced toxicities.

Group	Treatment	Sacrificed
Saline-treated control	*I.p.* daily dose of normal saline (0.5 mL) for 12 days	13th day
Extract control A	*Bv*RE for 6 days	7th day
Extract control B	*Bv*RE for 6 days	13th day
Ifosfamide control A	Ifosfamide for 6 days to induce toxicity	7th day
Ifosfamide control B	Ifosfamide for 6 days to induce toxicity	13th day
Combination	Both ifosfamide and *Bv*RE daily for 6 days	7th day
Curative	Ifosfamide for the first 6 days and then *Bv*RE for the next 6 days	13th day
Prophylactic	*Bv*RE for the first 6 days and then ifosfamide for the next 6 days	13th day

Ifosfamide (50 mg/kg/day) was administered intraperitoneally while *Bv*RE (1000 mg/kg/day) was administered orally, and all the animals were sacrificed on the 13th day of treatment.

### Biochemical estimation

For biochemical analysis, blood was collected directly from heart and serum was separated by centrifugation, and kits were used for the measurement of aspartate aminotransferase (AST), alanine aminotransferase (ALT), alkaline phosphatase (ALP), lactate dehydrogenase (LDH) and serum albumin (SA)^45,46^. Whole blood was used to find blood count, cholesterol, and triglycerides. The serum was separated after centrifugation Tissue homogenates (10% w/v) were prepared by homogenizing 500 mg of each tissue in 5 mL of 100 mM phosphate buffer (pH 7.4) and used for estimation of selected oxidative stress parameters including acetylcholinesterase (AChE, Ellman et al*.*), malondialdehyde (MDA, Ohkawa et al*.*), catalase (CAT, Hugo Aebi) and glutathione S-transferase (GSH, Yu et al*.*)^47–49^.

### Histopathological examination

Organs (kidney, liver, brain, and heart) were quickly removed after sacrificing the animals and were preserved in 10% formalin for histopathological examination. The tissues were embedded in paraffin wax, sectioned longitudinally and stained with hematoxylin and eosin for analysis under the microscope.

### MTT assay

The MTT (3-(4,5-dimethylthiazol-2-yl)-2,5-diphenyltetrazolium bromide) assay protocol used to find in vitro percentage viability of HeLa cells against ifosfamide, *Bv*RE, and their combination has already been reported in our previous publication^15^. The cells were treated with the selected concentrations (0.003, 0.03, 0.3, 30, and 60 µg/mL) of *Bv*RE, ifosfamide, and their combination to calculate percentage viability and dose–response curves were plotted to find *IC*_*50*_ values. The experiment was repeated in triplicate with two repeats for each concentration. So, the values represent the mean and standard deviation of six reading.

The following relation was used to evaluate the combined effect of *Bv*RE and ifosfamide on cytotoxicity:

Combination index = (Conc. of *Bv*RE in combination for *IC*_*50*_)/(*IC*_*50*_ of *Bv*RE) + (Conc. of ifosfamide in combination for *IC*_*50*_)/(*IC*_*50*_ of ifosfamide).

### Statistical analysis

Variations among groups and the significance of data were analyzed by using descriptive statistics and one-way analysis of variance (ANOVA) followed by post hoc Scheffe test on SPSS (ver. 13).

## Conclusion

The present study deduced that considerable toxicities were associated with the administration of ifosfamide (50 mg/kg/day, *i.p.*) in rats. A broad range of biochemical analyses and histopathological examination of organ tissues were performed to ascertain nephro, hepato, neuro, and cardiotoxic effects of ifosfamide. On the other hand, *Bv*RE played a significant ameliorative role in combination, prophylactic and curative regimens against ifosfamide-induced toxicities. However, prophylactic treatment of the extract exhibited relatively superior protective effects compared to the combination and curative administration of *Bv*RE. These findings suggest further investigation of the effects of *Bv*RE on ifosfamide-induced toxicity and anticancer activity in the xenograft mice model.
